# Are we fully exploiting genetic discoveries to understand and treat Alzheimer’s disease?

**DOI:** 10.1007/s00335-026-10227-2

**Published:** 2026-04-21

**Authors:** Julie Williams

**Affiliations:** Moondance Dementia Research Laboratory, UK Dementia Research Institute, Cardiff University, Hadyn Ellis Building, Cardiff, CF24 4HQ UK

Discovering relationships between clinical diseases and variation in DNA provides connections to putative disease pathways and novel targets for future therapies. This genetic strategy is and will continue to shape our understanding of disease mechanisms, epidemiology, advance clinical trials and drug discovery and revolutionise therapeutic development. Over the last decade genome-wide association studies (GWAS) have driven most of these advances for common diseases with genetic components (Sims et al. 2020). The effects of human genetic variability combined with the small individual effect sizes of disease associations commonly observed, have been overcome by powerful GWAS studies with stringent levels of proof, underpinned by large international collaborations with in-built replication across different laboratories and countries. These studies have been particularly informative for brain diseases, of which Alzheimer’s disease is a prime example. This commentary will provide a brief overview of the genetics of Alzheimer’s disease, illustrate how this can inform disease mechanisms and treatments and review whether we are exploiting the full potential of genetic discoveries, highlighting the failure of pharmaceutical companies to harness the wealth of genetic knowledge in clinical trials, for example.

The heritability of Alzheimer’s disease (AD) is estimated as 58–79% for late onset AD (<65 years) and over 90% for early onset AD (>65 years) (Sims et al. 2020). Early genetic findings from family studies of rare forms of predominantly early onset AD, discovered relationships between mutations in three genes, *APP* and *presenilin1 & 2*. The functions of these genes coding for amyloid-β and its processing, focussed thinking on the activity of amyloid plaques, first observed by Alois Alzheimer over a century ago. In 1992 John Hardy and colleagues (Hardy and Higgins 1992) proposed the Amyloid Cascade Hypothesis, which posited amyloid plaques as the main cause of Alzheimer’s disease (AD). This hypothesis dominated research for decades and gave rise to several treatments which have successfully removed amyloid plaques from the brains of AD cases. However, these treatments have failed to stop the disease but have showed slower progression in a proportion of individuals, although differentiating these individuals is challenging without detailed information including genetics (van Dyck et al. 2023; Sims et al. 2023).

In 1993 the apolipoprotein E (APOE) genetic locus was identified as a strong risk factor for the more common form of AD with an onset at 65 years or later (Sims et al. 2020). The APOE risk isoforms (E2,3,4) are now known to affect a wide range of molecular and cellular functions in several cell types (Jackson et al. 2024). These include, neuroinflammation, energy metabolism and blood-brain permeability, as well as links to amyloid-β processing. Over the last decade GWAS of AD have identified around 90 genes which confer susceptibility and these findings show a more complicated and diverse aetiology (ADGC et al. 2025). GWAS studies have not been without challenges. Many of the loci implicated are in non-coding regions of the genome where the functional variant/s is ambiguous. However, triangulating these findings with other genomic evidence for example, have clarified relationships in many cases.

From the earliest GWAS of AD it became clear that the immune system plays a significant role or roles in late onset AD (Harold et al. 2009, Jones et al. 2015; Lambert et al. 2009; see Sims et al. 2020). In addition, a disproportionate number of susceptibility genes for AD are expressed in microglia (Sims et al. 2017), including coding variants in *TREM2* (Triggering receptor expressed on myeloid cell 2) and *PLCG2* (phospholipase C gamma 2), which share a similar functional pathway. The TREM2-PLCγ2 pathway recognises amyloid-β and anionic lipids and mediates microglial metabolic reprogramming, survival and activation programmes. It facilitates the preservation of synaptic integrity, activation of microglia by amyloid-β, their clustering around plaques, plaque compaction and clearance of amyloid and debris (Heneka et al. 2025). The first described TREM2 variant R47H associated with Alzheimer’s disease risk (Guerreiro et al. 2013; Jonsson et al. 2013; see Sims et al. 2020) is hypofunctional and associated with defective ligand binding (e.g. APOE and anionic ligands). Downstream of TREM2, the PLCG2 associated variant (P522R) protects against Alzheimer’s disease, and is hyper-functional (Magno et al. 2019; Maguire et al. 2020). Hence, the functional activity of the pathway associates with cell survival, the level of activation and endocytosis and amyloid- β response, with specific alterations in ligand responses. The bidirectional impact of modulating TREM2-PLCγ2 axis highlights how microglia can be shifted between detrimental and protective roles and the pathways potential tractability as a therapeutic target. Several genetic associations also implicate the role of the complement system in AD, including the genes *CLU* encoding clusterin, and *CR1* encoding complement receptor 1 (Sims et al. 2020). Complement is a critical component of the innate immune system, an inflammatory system evolved to remove dead/dying cells and invading pathogens. Clusterin (CLU) AD risk alleles are associated with reduced clusterin protein and heightened inflammatory profiles mediated by astrocyte reactivity and microglia effects on synaptic integrity (Lish et al. 2025). There are already moves to modify current complement drugs to cross the blood-brain barrier to treat AD. It is interesting that there is also an association with the human leucocyte antigen (HLA) genetic locus, implicating HLA biology and T-cell responses in the development of AD. It is noteworthy that significantly more genes than expected by chance also implicate the role/s of endocytosis and cholesterol/lipid processing (Sims et al. 2020). All these lines of evidence strongly support significant immune components in the development of AD. It is also now clear that multiple disease mechanisms contribute to AD. Moreover, even single AD genetic risk variants affect multiple disease processes. We articulated this idea in the Multiplex Model of AD (Sims et al. 2020), emphasising that AD is dependent on the combined changes in several biological pathways and that our understanding and treatment of this disease must broaden its focus from single causes, such as the action of amyloid plaques alone.

Discovering genes which influence AD has also allowed the identification of those susceptible. This is important in a disease which begins up to 20 years before dementia symptoms are observed (Sims et al. 2020). In 2015 we developed a polygenic risk score which has an accuracy of around 80% to predict AD in neuropathologically confirmed AD case-control samples (Sims et al. 2020). However, population-based estimates may be lower due to control bias (well individuals who later develop AD) and survival bias (AD risk factors associated with early death). Polygenic risk scores developed from predominantly European population samples have proved informative across several other populations, although more diversity has yet to be tested and population specific polygenic scores calculated (Nicolas et al. 2025). Identifying those at greater risk of developing AD will enable the targeting of preventative therapies, as they emerge, clarifying diagnosis and facilitating other forms of research, such as clinical trials, epidemiology, neuropathology, neuroimaging, biomarker and drug development. It is therefore surprising that some of these research areas fail to exploit the full power of these genetic discoveries. Notably, few if any, clinical trials of new therapies for AD, test individuals’ polygenic AD risk, even though this is easily achieved. Once sampled, a simple genomic typing can inform both overall genetic risk for AD and risk for specific disease risk pathways. Matching individual therapies to those showing biological risk to the disease pathway/s targeted, can only improve success rates for clinical trials. So why don’t pharmaceutical companies exploit these discoveries?

Similarly, biomarker discovery research also fails to include polygenic susceptibility in their designs. Testing for biological signs of active disease in those at genetic risk could increase power to detect biomarkers. However, recent major advances in AD biomarkers are beginning to integrate genetics. The discovery of plasma p-tau and its approval as the first blood-based biomarker for AD, provides opportunities to combine the complementary strengths of genetic predisposition and biomarkers in diagnosis and disease staging (Palmqvist et al. 2025). However, epidemiology does better as many cohort studies already include extensive genetic data, thus allowing the testing of integrated environmental and genetic relationships. Research integrating genetics with other transcriptomic, proteomic and neuropathological data have also used the power of genetics, especially when exploiting new technologies such as single-cell spatial transcriptomics and Artificial Intelligence (AI) in analytics. All these approaches are uncovering novel relationships, expanding our understanding of this complex disease.

Genetic data can also underpin the development of better models of the more common forms of AD, through the creation of human induced pluripotent stem cells (hiPSC) sampled from individuals with different genetic risk loadings and profiles (Maguire et al. 2025). For example, we have established over 100 hiPSC lines sampled from a cohort of over 6000 AD cases and controls, who have either extreme high or low polygenic risk for AD (early or late onset), which show significant effects of polygenic risk on a variety of microglial processes. These hiPSC models can be transformed into various cell types, tested in vitro or in vivo, for example, through mouse chimeras and selected or modified to reflect different potential disease pathways. We have already observed significant differences between high and low AD polygenic risk in several independent microglial functions. Thus, opening new areas of research for novel disease mechanisms and targets and providing a human platform for therapeutic testing for both gene and drug therapies for the most common form of AD.

Let’s be clear, we are at the early stages of understanding how AD susceptibility genes influence the disease, but the potential for future therapeutic development is significant. Only a proportion of genetic evidence has been explored for disease mechanisms and therapeutic targets and there are more genetic relationships to find, specifically testing relationships using long-read sequencing interrogating the dark genome (major part of the genome which is difficult to assay by current techniques but includes variants which have biological effects). There is also more to be achieved using current genetic findings in clinical trials, diagnosis, epidemiology, biomarker discovery and in developing better human-based models of disease. Discovering genetic associations with AD has connected clinical disease with disease mechanisms with compelling evidence and at a level at which treatments can be designed. In fact, it is difficult to justify not using genetics to inform future AD research to underpin the discovery of new disease mechanisms, therapeutic targets and precision medicine.

## Data Availability

No datasets were generated or analysed during the current study.
